# Phytochemicals as Potential Epidrugs in Type 2 Diabetes Mellitus

**DOI:** 10.3389/fendo.2021.656978

**Published:** 2021-06-01

**Authors:** Karina Ramírez-Alarcón, Montserrat Victoriano, Lorena Mardones, Marcelo Villagran, Ahmed Al-Harrasi, Ahmed Al-Rawahi, Natália Cruz-Martins, Javad Sharifi-Rad, Miquel Martorell

**Affiliations:** ^1^Department of Nutrition and Dietetics, Faculty of Pharmacy, University of Concepción, Concepción, Chile; ^2^Department of Basic Science, Faculty of Medicine, Universidad Catolica de la Santisima Concepcion, Concepción, Chile; ^3^Scientific-Technological Center for the Sustainable Development of the Coastline, Universidad Catolica de la Santisima Concepcion, Concepción, Chile; ^4^Natural and Medical Sciences Research Centre, University of Nizwa, Birkat Al Mouz, Oman; ^5^Faculty of Medicine, University of Porto, Alameda Prof. Hernâni Monteiro, Porto, Portugal; ^6^Institute for Research and Innovation in Health (i3S), University of Porto, Porto, Portugal; ^7^Laboratory of Neuropsychophysiology, Faculty of Psychology and Education Sciences, University of Porto, Porto, Portugal; ^8^Phytochemistry Research Center, Shahid Beheshti University of Medical Sciences, Tehran, Iran; ^9^Facultad de Medicina, Universidad del Azuay, Cuenca, Ecuador; ^10^Centre for Healthy Living, University of Concepción, Concepción, Chile; ^11^Universidad de Concepción, Unidad de Desarrollo Tecnológico, UDT, Concepción, Chile

**Keywords:** type 2 diabetes mellitus, hyperglycemia, protein target, epigenetic, epidrug, phytochemicals

## Abstract

Type 2 diabetes Mellitus (T2DM) prevalence has significantly increased worldwide in recent years due to population age, obesity, and modern sedentary lifestyles. The projections estimate that 439 million people will be diabetic in 2030. T2DM is characterized by an impaired β-pancreatic cell function and insulin secretion, hyperglycemia and insulin resistance, and recently the epigenetic regulation of β-pancreatic cells differentiation has been underlined as being involved. It is currently known that several bioactive molecules, widely abundant in plants used as food or infusions, have a key role in histone modification and DNA methylation, and constituted potential epidrugs candidates against T2DM. In this sense, in this review the epigenetic mechanisms involved in T2DM and protein targets are reviewed, with special focus in studies addressing the potential use of phytochemicals as epidrugs that prevent and/or control T2DM *in vivo* and *in vitro*. As main findings, and although some controversial results have been found, bioactive molecules with epigenetic regulatory function, appear to be a potential replacement/complementary therapy of pharmacological hypoglycemic drugs, with minimal side effects. Indeed, natural epidrugs have shown to prevent or delay the T2DM development and the morbidity associated to dysfunction of blood vessels, eyes and kidneys due to sustained hyperglycemia in T2DM patients.

## Introduction

Type 2 diabetes Mellitus (T2DM) is a metabolic disorder associated with high morbi-mortality rates and expenses in the healthcare system at worldwide level (1). Featured by an insulin deficit triggered by the pancreatic dysfunction of β-type cells and insulin resistance in target organs (2), in 2014, the World Health Organization (WHO) referred that 8.5% of adults (18 years or older) had this disease, and that in 2015, 1.6 million people died as a direct result of T2DM. Even high blood sugar levels alone has been the cause of another 2.2 million deaths in 2012 (3).

The global increase in obesity, along with physical inactivity and energy dense diets (4), has triggered direct or epigenetic changes in phenotype that would be the cause of the disease (5). So, these environmental changes have been correlated with the large increase in the number of T2DM patients. Recently, it has been recognized that T2DM results from regulatory imbalances at both genetic and epigenetic levels. Currently it is known that epigenetics has an important role in the insulin secretion and action and T2DM development (6). The differentiation of β-pancreatic cells is controlled by several genes, such as GLP1 (glucagon-like peptide-1; stimulates insulin secretion and inhibits glucagon secretion), PAX4 (paired box gene 4; involved in pancreatic islet development) and PDX1 (pancreatic and duodenal homeobox 1; involved in pancreatic development, β-cell differentiation and the maintenance of mature β-cell function) receptor, with all these genes being regulated at the epigenetic level. In addition, some factors involved in insulin resistance, such as the nuclear factor kappa-B (NF-κB), osteopontin and Toll-like receptors, are also epigenetically regulated (7). Human T2DM case-control studies and intervention studies in non-diabetic people showed epigenetic alterations of PDX1, CDKN1A (cyclin-dependent kinase inhibitor 1; involved in cell cycle regulation) and GLRA1 (glycine receptor alpha 1; down-regulation of neuronal excitability) genes which seem to contribute to diabetes (6). An increase in the level of DNA methylation of PDX-1 has been related to a reduced activity in pancreatic islets and a dysregulation of pancreatic β-cells in T2DM (8). CDKN1A overexpression decreases insulin secretion and proliferation (9) and GLRA1 silencing in clonal β-cells reduced glucose-stimulated insulin secretion (10). In addition, physical activity alter DNA methylation of T2DM gene candidates such as FTO (fat mass and obesity-associated protein; associated with energy intake) and TCF7L2 (transcription factor 7-like 2; blood glucose homeostasis) in adipose tissue (6). In obese humans after a metabolic surgery, epigenetic and metabolic changes were reported in skeletal muscle during the improvement of insulin sensitivity (11). The major sign of uncontrolled diabetes is hyperglycemia, and when it is maintained for prolonged periods, results in the blood vessels destruction, that consequently triggers damages to the heart, eyes, kidneys and central nervous system (CNS) (12). As a result, both macrovascular (atherosclerotic) and microvascular (retinopathy and nephropathy) disorders occur. These complications are the main causes of mortality in diabetic patients. In this sense, intensive lifestyle modification, pharmacotherapy or both have been proposed, as they are able to reverse or delay the T2DM complications (2). Studies have reported an increased use of natural products in patients with T2DM (13–15); this is due to the long-term use of oral hypoglycemic agents and insulin, characterized by numerous side effects, that include episodes of hypoglycemia, gastrointestinal problems (nausea, vomiting and diarrhea), edema and even hepatorenal disorders (16, 17). The therapeutic activity of these plants, used as food or infusions, depends on the interaction of several kinds of phytochemicals. Actually, there are more than 1,200 species of medicinal plants with antidiabetic activity, and of these approximately 200 pure bioactive compounds possess potent hypoglycemic properties (18) and have an important role in histone modification and DNA methylation (19). In this sense, here we aim to discuss the epigenetic mechanisms involved in diabetes and protein targets, also giving a special emphasis to *in vitro* and *in vivo* studies and clinical trials, addressing the phytochemicals with epidrug potential in diabetes.

## Epigenetic Mechanisms of Type 2 Diabetes Mellitus

The hyperglycemia occurring in T2DM is a systemic alteration that affects all tissues leading to long-term diseases (20). Particularly, hyperglycemia is able to alter the expression of genes involved in insulin resistance, low grade systemic inflammation and renal fibrosis (21). The biological process underlying the changes in gene expression comprises the epigenetic regulation of the genome of different tissues including skeletal muscle, liver, pancreas, blood and adipose tissue for T2DM (22). The physical basis of the epigenetic regulation are changes in the chromatin structure without changes in DNA sequence, some of them could even be transmitted through generations. Globally, the epigenetic modifications can be grouped into three categories: DNA methylation, posttranslational histone modifications and non-coding RNAs (23).

### General Epigenetic Mechanisms

Methylation of DNA was the first epigenetic mechanism discovered and is associated to transcriptional genes silencing, whose promoter has been methylated. It consists in the covalent attachment of a methyl group at the 5’ carbon of cytosine residues in a promoter region rich in cytosine-phosphate-guanine (CpG), called CpG islands (24). The presence of these modified nucleotides is able to recruit methyl-CpG binding proteins that promote chromatin condensation and, therefore, restrict the accessibility of transcription factors and the general transcription machinery to the promoter (24). The DNA methyltransferases (DNMTs) are a family of enzymes that catalyze the methyl transfer from S-adenosylmethionine (SAM) to cytosine and are composed of DNMT1, DNMT3A and DNMT3b. The removal of the methyl group is accomplished by demethylases of the ten eleven translocation (TET) family, which mediates an oxidation of the methyl group that renders a 5-hydroxymethylcytosine that is later replaced by cytosine during DNA repair. DNA demethylation counteracts the chromatin compaction elicited by methyl marks and is usually linked to transcriptional activation (25). DNMT3A was reported as epigenetic mediator of adipose insulin resistance in mouse and human adipocytes (26).

Post-translational histone modifications are a diverse group of covalent modification generally located in the N-terminal. Briefly, the acetylation, methylation, phosphorylation and ubiquitination of specific residues are among the most studied, but there is an emerging number of up to 67 newly identified histone modifications with the potential of regulating gene expression (27). First, histone acetylation occurs in lysine residues and activates gene transcription through chromatin de-condensation and acts as a binding site for transcriptional activators. Histone acetyl transferases (HAT) catalyze acetylation and so stimulate gene transcription, whereas histone deacetylases (HDACs) remove the acetyl group from histones, promoting the transcriptional repression (28). Methylation of histones takes place in lysine and arginine residues and each residue could present different methylation states, including mono, di and trimethyl lysine, while arginine may be symmetric or asymmetrically mono or dimethylated. Depending on the specific residue and the methylation state, transcription effect could be activated or repressed. For example, the mono, di or trimethylation of histone H3 (H3) at Lys4 (H3K4m1/2/3) have been associated with transcriptionally active genome regions, while trimethylation of H3 at Lys9 or at Lys27 (H3K9m3/H3K27m3) and trimethylation of H4 at Lys20 (H4K20m3) are abundant in silenced DNA regulatory elements (29). Second, histone methylation is carried out by histone methyltransferases (HMTs) of different specificity for lysine or arginine, with methyl removal depending on the action of demethylases with defined specificity as well. Other emerging histone modifications relevant for T2DM are crotonylation and β-hidroxybutyrilation, both affecting lysine residues (30, 31). Finally, gene expression can also be regulated transcriptionally and post-transcriptionally by non-coding RNA molecules. Among them, miRNAs are typically composed of 21 to 23 nucleotides long and mediate post-transcriptional repression through binding to complementary regions of specific target mRNAs, leading to its degradation by the RISC complex (32). Another relevant non coding RNA for T2DM are long non-coding RNAs (lncRNAs) involved in recruitment of DNMTs and histone modifiers to their target genes and, particularly the eRNAs, facilitate promoter-enhancer looping, thus increasing the transcription rate of neighboring genes (33).

### Epigenetic Alterations in T2DM

#### DNA Methylation

T2DM is a complex metabolic disease in which many interconnected pathologic mechanisms ensue. Initial studies analyzed DNA methylation of candidate genes for T2DM such as INS (insulin), PDX1, PPARGC1A (PGC1α; transcriptional co-activator), and GLP1R (GLP1 receptor) in human pancreatic islets from donors with TD2M and non-diabetic controls (22, 34, 35). In these studies, islets from T2DM donors were found to have increased DNA methylation and decreased expression of these key genes associated with impaired insulin secretion (22). In addition, the increase of DNA methylation of these genes seemed to directly be associated with high glucose and glycated hemoglobin (HbA1c) levels. In pancreatic islets, early molecular alterations occur and mediate islet dysfunction before the onset of diabetes (36). DNA methylation patterns of β-cell are dynamic during maturation and T2DM onset and evolution (37). Some differentially methylated regulatory elements have been associated with key function genes such as PDX1, TCF7L2 and NKX6-1 (homeobox protein Nkx-6.1; regulation of islet transcription factors and genes involved in glucose and insulin homeostasis). In Langerhans Islets from obese mice which differed in their degree of hyperglycemia and in liver fat content a semi-explorative approach identified 497 differentially expressed and methylated genes linked to insulin secretion and extracellular matrix-receptor interaction (38). In addition, the comparison of mouse data with DNA methylation levels of TD2M participants of European Prospective Investigation Cancer (EPIC)-Potsdam cohort revealed 105 genes with altered DNA methylation at 605 CpG sites, which were associated with future T2DM. The first epigenome-wide association studies of DNA methylation markers of obesity and T2DM are helping to gain a comprehensive insight into the global epigenetic alterations related to the onset of T2DM. In samples of blood cells of 5,387 individuals, changes in methylation markers of genes involved in lipid metabolism, substrate transport and inflammatory pathways have been reported (39). In addition, although these changes have been detected in a non-metabolically relevant tissue, similar DNA methylation abnormalities were also detected in adipose, muscle and liver tissue samples of a small subset of participants. Interestingly, the DNA methylation disturbances induced by obesity can predict future development of T2DM (22, 39). Another study conducted in the same population, developed a prospective nested-control study that analyzed genome-wide DNA methylation markers in blood cells of participants before the T2DM onset. DNA methylation markers at five genes: ABCG1 (ATP-binding cassette sub-family G member 1; regulates cellular lipid homeostasis, including pancreatic β cells), PHOSPHO1 (phosphatase, orphan; involved in bone mineralization), SOCS3 (suppressor of cytokine signaling 3; negative regulator of insulin signaling), SREBF (sterol regulatory element binding transcription factor 1; regulator of hepatic lipogenesis) and TXNIP (thioredoxin interacting protein; key component of pancreatic β cell biology, nutrient sensing, energy metabolism and regulation of cellular redox) have been associated with up to four times higher risk of future T2DM (40), so that these markers raise the possibility to identify normoglycemic subjects that could benefit from early pharmacological or lifestyle interventions to prevent the T2DM development (40). Besides these genomic-wide studies of DNA methylation markers in T2DM, several other studies have focused on the epigenetic markers of specific genes in the context of particular pathogenic events related to T2DM. In this regard, chronic inflammation has been recognized as a key pathologic feature of T2DM, accompanied by DNA methylation changes. In blood mononuclear cells from T2DM patients, the methylation level at the CpG sites of the monocyte chemoattractant protein (MCP)-1 promoter is decreased, and associated to an overexpression of this chemotactic factor in serum (41). A similar decreased in DNA methylation was found at interleukin (IL)-1β promoter in blood cells and at 3’UTR of TXNIP in skeletal muscle from T2DM patients (42, 43). In other study, increased methylation and decreased expression of PPARGC1A were reported in skeletal muscle from TD2M humans and when this gene was silenced in human islets, insulin secretion was decreased (44).

#### Histone Tail Modifications

Alterations in the multiple types of histone modifications have also been implicated among the epigenetic mechanisms underlying T2DM inflammation. Studies mainly developed in cell models have reported changes in histone marks within regulatory elements of inflammation genes in response to hyperglycemia. For example, Miao and Gonzalo (45) found an increase of histone acetylation at NF-κB promoter in THP-1 monocytes induced by a transient exposure to high levels of glucose. The higher acetylation was correlated to increased recruitment of p300/CBP-associated factor (PCAF) to NF-κB promoter and overexpression of proinflammatory target genes, like cyclooxygenase-2 (COX-2) and tumor necrosis factor (TNF) (45). Interestingly, similar changes in histone modification of NF-κB have been stated in monocytes from T2DM subjects, showing an *in vivo* correlate of the epigenetic changes (45). IL-8 is another proinflammatory gene whose overexpression is induced by hyperglycemia in human primary vascular cells by an epigenetic mechanism involving hyperacetylation of its promoter region (46). Ibarra Urizar and Prause (47) hypothesized that prolonged exposure of β-cells to IL-1β induce β-cell dedifferentiation characterized by impaired glucose-stimulated insulin secretion, reduced expression of key β-cell genes and changes in histone modifications. In their study they observed that IL-1β at low concentration induces epigenetic changes associated with loss of β-cell identity as observed in T2DM. The epigenetic effect of hyperglycemia is not limited to induction of hyperacetylation but also triggers H3K4 hypermethylation and H3K9 hypomethylation in NF-κB p65, leading to a persistent upregulation in endothelial cells under hyperglycemic conditions. These changes in histone methyl marks are mediated by the recruitment of SETD7 (SET domain containing 7, histone lysine methyltransferase) and lysine-specific demethylase 1 (LSD1) to NF-κB promoter and are linked to a higher expression of NF-κB regulated genes, like MCP-1 and vascular cell adhesion protein 1 (VCAM) (48). In addition, the *ex vivo* methylation changes of NF-κB p65 in endothelial cells have also been shown in blood mononuclear cells of T2DM patients, where the unbalance is also related to an increased expression of MCP-1, intercellular adhesion molecule (ICAM)-1 and COX-2 (49). Similarly, the increased expression of IL-8 in endothelial cells is not only mediated by higher histone acetylation but also by H3H4 hypermethylation induced by SETD7 methylase (50). These examples underline that hyperglycemia induce an overexpression of proinflammatory genes by redundant epigenetic histone marks, that in the case of NF-κB, additionally involve acetylation of lysine residues of transcription factor itself (51). Other relevant overexpression states of proinflammatory genes mediated by methylation changes related to hyperglycemic stimulus are the IL-6 and MCP-1 in vascular smooth muscle cells, IL-6 in rat cardiomyocytes, IL-12 subunit beta (IL-12B), macrophage inflammatory protein (MIP)-1α, MIP-1β, and IL-6 in THP-1 monocytes (52).

#### Non-Coding RNA

Post-transcriptional repression mediated by miRNA also contribute to the dysregulation of proinflammatory gene expression in T2DM, usually mediating the accelerated mRNA decay of transcriptional activators of inflammatory mediators. An increased expression of miR-125b in vascular smooth muscle cells has also been reported in a T2DM mouse model (53). The higher levels of miR-125b triggers a decrease in the SUV39H1 histone methylase levels, leading to a reduction of the H3K9 activating signal in the promoters of IL-6 and MCP-1 (53). Other miRNAs upregulations in T2DM impacting the proinflammatory genes expression are: miR-146a decreasing the expression TNF receptor-associated factor 6 (TRAF6) and interleukin 1 receptor-associated kinase 1 (IRAK1); miR-200c family members increasing the expression of COX-2 and MCP-1; and miR-504 increasing the expression of IL-6, COX2 and MCP-1 (52). Abnormalities of lncRNA have also been observed in T2DM-associated inflammation. For example, the E33 lncRNA levels were increased in macrophages of a mouse model of T2DM as well as the human homolog MIR143HG (54, 55). However, only partial data is now available related to the mechanism of deregulation, but an induction of several proinflammatory genes, like IL-6, TNF and COX-2, whereas the downregulation of MCP-1 and of anti-inflammatory IL-10 has been reported (55). Sathishkumar and Prabu (56) related that metastasis-associated lung adenocarcinoma transcript 1 (MALAT1) is another lncRNA that may regulate the T2DM-related inflammation through upregulation of serum amyloid antigen (SAA), ultimately inducing IL-6 and TNF in human umbilical vein endothelial cells (HUVECs).

## Phytochemical Regulation of Protein Targets for the Treatment of Diabetes

The treatment of T2DM is usually focused in the regulation of protein target activities (7). Several phytochemicals from fruits, vegetables, spices, teas, and medicinal plants may regulate epigenome and exhibit low toxicity in chronic administration (57). The applicability of major phytochemical groups such as polyphenols, terpenoids, organosulfur and alkaloids as epidrugs is experimentally established (58, 59). In this section, the common protein targets for T2DM treatment and phytochemicals that modulate the activities of these proteins with antidiabetic activities in *in silico*, *in vitro* and *in vivo* studies are presented, while data related to clinical trials is shown in **Table 1**.

**Table 1:** Clinical trials related to natural epidrugs to treat Type 2 Diabetes Mellitus.

Supplement	Period	Population	N (T/C)	Results	References
11β-HSDs inhibitors					
Ginger					
capsule (1.6 g/d)	12 weeks	T2DM 30–60 yr BMI 20–35	33/30	↓FBG, HbAb1, INS, HOMA-IR	(60)^RCT^
tablet (2 g/d)	8 weeks	T2DM 30–70 yr BMI × 30	28/30	↓INS HOMA-IR	(61)^RCT^
capsule (3 g/d)	12 weeks	T2DM 30–70 yr BMI <40	40/41	↓FBG, HbAb1	(62)^RCT^
capsule (1 g/d)	12 weeks	T2DM 20–60 yr BMI <30	39/31	↓FBG, HbAb1, INS, HOMA-IR	(63)^RCT^
tablet (2 g/d)	12 weeks	Women 18–45 yr BMI 30–40	39/31	↓FBG	(64)^RCT^
tablet (2 g/d)	12 weeks	Women 18–45 yr BMI 30–40	39/31	↓INS, HOMA-IR	(65)^RCT^
capsule (1 g/d)	10 weeks	Dialyzed 29–79 yr	18/18	↓FBG	(66)^RCT^
**Flavonoids, isoflavones and lignans**					
soy products (9 g/d protein)	1 yr	Women 40–70 yr BMI >25	323/39,062	↓glycosuria	(67)^CSS^
10 mg/d s-equol	12 weeks	Men and women 59.4 yr BMI ≥25	49/49	↓HbA1c	(68)^RCT,DB,Pl,CO^
tablet 54 mg/d genistein	2 yr	Women 49–67 yr BMI 25	198/191	↓FBG, INS, HOMA-IR	(69)^RCT,DB,PL^
360 mg/d lignan	2 × 12 weeks wash-out 8 weeks	T2DM Men and women 50–79 yr	37/36	↓HbA1c	(70)^RCT,DB,Pl,COS^
40 or 80 mg/d isoflavones	1 yr	Women 48–62 yr	68/68/67	↓FBG	(71)^RCT,DB,PL^
stratified phytoestrogens intake	2 yr	Men 47–83 yr	468	Lignan ↓C peptide Isoflavones no effect	(72)^CSS,PS^
stratified soy food intake	5.7 yr	Men and women 45–74 yr	43,176	↓T2DM risk isoflavones and unsweet soy	(73)^LS^
stratified soy foods	4.6 yr	Women 40–70 yr	64,191	↓T2DM risk	(74)^LS,CCS,PS^
stratified soy food intake	14 yr	Men and women 45–75 yr	75,344	Modest ↑T2DM risk BMI ≥25	(75)^CCS,PS^
stratified soy products and isoflavones	5 yr	Men and women 40–69 yr	59,791	↓T2DM risk in women BMI ≥25	(76)^CCS,PS^
stratified flavonoids and lignans intake	12 months	Men and women	15,258	↓T2DM risk	(77)^CCS,PS^
stratified isoflavones intake	2 yr	Pregnant women 28 yr	299	↓FBG, INS, HOMA-IR	(78)^CSS^
stratified genistein intake	1 yr	Women 45–74 yr BMI 26	255/107/46	↓INS	(79)^PL,DB,RCT^
stratified lignan intake	6 yr	Women 32–79	1,107/1,107	↓T2DM risk	(80)^LS^
**Curcumin**					
nano-micelles 30 mg/d	3 months	T2DM in treatment >18 yr	35/35	↓FBG,HbA1c	(81)^RCT,DB^
capsule 1,5g/d	9 months	Prediabetic ≥35 yr	120/120	↓T2DM risk, FBG,HbA1c, HOMA-IR, C-peptide ↑HOMA-β	(82)^RCT,DB^
500 mg/d plus piperine 5 mg/d	3 months	T2DM 18–65 yr	50/50	↓FBG, C-peptide	(83)^RCT,DB^
1.5 g/d ethanolic extract	6 months	T2DM ≥35 yr	107/106	HOMA-IR	(82)^RCT,DB,PL^
nano-micelles 300 mg/d	3 months	T2DM 18–65 yr BMI≥24	50/50	↓ FBG,HbA1c, HOMA-IR	(84)^RCT,DB^
**Black and green tea**					
3 cups/d green tea	14 weeks	35–65 yr	65/58	No changes	(85)^RCT,^
1 g/d green tea	12 weeks	T2DM 37–78 yr	32/28	↓FBG	(86)^RCT,DB^
1 capsule 200 mg green tea extract twice daily 9 months plus thrice 9 months	18 month	T2DM 20–45 yr	17/14	No changes	(87)^RCT,DB^
3 cups/d black tea	12 week	T2DM 44–55 yr	30/20	↓HbA1c	(88)^RCT^
capsule 560 mg polyphenols/d	20 weeks	T2DM >18 yr	27/28	No effect	(89)^RCT,DB^
**Epigallectine-3-galleate**					
capsule 150 mg/d	8 weeks	T2DM 20–60 yr	25/25	↓FBG	(90)^RCT,DB^
**PTP1B inhibitors**					
**Resveratrol**					
capsule 10 g/d	4 week	T2DM men >18 yr	10/9	↓HOMA-IR	(91)^PL,DB,RCT^
capsule 250 mg/d	3 months	T2DM in treatment 30 × 70 yr	28/29	↓HbA1c	(92) ^LS,RCT^
75 mg/d	12 week	women × 59 yr BMI <30	15/14	No effect	(93)^PL,DB,RCT^
capsule 1,500 mg twice daily	8 week	NALD men BMI >25	10/10	No effect	(94)^PL,DB,RCT^
capsule 300 mg/d	4 week	men 18–70 yr BMI >30	12/12	No effect	(95)^PL,DB,RCT^
Mediterranean diet	2 yr	T2DM 55–80 BMI ≥30	1,000 (quintiles)	↓FBG	(96)^PL,DB,RCT^
**17β-HSD inhibitors**					
**Extra virgin olive oil**					
500 mg/d OLF	12 weeks	Men 35–55 yr BMI 25–30	21/22	↑β-cell function, ↓FBG, INS sensitivity	(97) ^RCT,COS^
500 mg/d OLF	14 weeks	T2DM 18–79 yr BMI <40	41/38	↓HbA1c, fasting INS	(98)^RCT^
20 ml HPOO 20 ml LPOO	6 weeks	18–75 yr	34/9 29/9	↓FBG	(99)^PL,RCT^
Mediterranean diet with 50 ml/d EVOO	5 yr	55–80 yr	1,154/1,240	↓ T2DM risk	(100)^PL,RCT^
**GFAT inhibitors**					
**Fenugreek**					
capsule 2.5 g twice daily	1 weeks	T2DM and CAD	30/30	↓FBG, 2 h glucose	(101)^PL^
capsule 1.176 g/d	6 weeks	Overweight men × 18–59 yr	20/20	↓FBG, fasting INS	(102)^PL,RCT^
capsule 6.3 g/d	12 weeks	T2DM in treatment 25–65 yr	46/25	↓FBG, HbA1c, 2 h glucose	(103)^PL,RCT^
capsule 1 g/thrice daily	12 weeks	T2DM in treatment 30–70 yr	30/30	↓FBG, post prandial glucose, HbA1c	(104)^PL,RCT^
powder 25 g/d	24 weeks	T2DM 30–70 yr	60/10	↓glucosuria, HbA1c	(105)

BMI, body mass index (kg/m^2^); CAD, coronary arterial disease; CCS, case-cohort study; COS, cross-over study; CSS, cross-sectional study; DB, double-blind; FBG, fast blood glucose; T2DM, type 2 Diabetes Mellitus; HbA1c, glycosylated hemoglobin; INS, insulin; IR, insulin resistance; HOMA, Homeostasis Model Assessment; HPOO, high phenolic extra virgin olive oil; LPOO, low phenolic olive oil; LS, longitudinal study; NALD, no-alcoholic liver disease; OLF, olive leaf extract; PL, parallel trial; PPG, postprandial glucose; PS, prospective study; RCT, randomized clinical trial; T/C, treated v/s control.

### Decrease Insulin Resistance *via* 11β-Hydroxysteroid Dehydrogenase Inhibition

Cortisol plays an important role in metabolism and T2DM, and the inhibition of the hepatic glucocorticoid receptor was found to reduce the serum glucose levels in T2DM mice while improve the insulin resistance (106). Indeed, an abnormal glucocorticoid metabolism has been linked to T2DM (107). Specifically, the 11β-hydroxysteroid dehydrogenase (11β-HSDs) is an oxidoreductase enzyme that catalyzes the conversion of inert 11 keto-products (cortisone, 11-dehydrocorticosterone) to active glucocorticoids (corticosterone, cortisol). 11β-HSD1 regulates cortisol levels mainly in adipose, hepatic and brain tissues (108). Briefly, 11β-HSDs has several isoforms in humans, where 11β-HSD1 is a NADPH-dependent isoform highly expressed in key metabolic tissues of liver, adipose, pancreas, and skeletal muscle (109), while 11β-HSD is a potent therapeutic target whose inhibition may be useful for the treatment of T2DM (107, 110).

A high enzyme activity of 11β-HSD1 is related with the development TD2M and others metabolic disorders and the inhibition of this enzyme attenuate the development of T2DM, insulin resistance, metabolic syndrome and other diseases mediated by excessive cortisol production (108).

Zhu and Ge (111) in a study compared the ability of flavonoids and isoflavonoids to inhibit human 11β-HSD1 and 11β-HSD2. Briefly, the authors found that apigenin, quercetin, genistein and (±)-equol were able to inhibit human 11β-HSD1, with IC_50_ values of 2.2, 5.4, 11.0, and >100 μM, respectively. However, apigenin and (±)-equol were not able to inhibit 11β-HSD2 at doses as high as 100 μM, while genistein and quercetin inhibited it by 60 and 50% at 100 μM, respectively (111). In streptozotocin–nicotinamide induced diabetic rats, quercetin showed antidiabetic activity acting as 11β-HSD1 inhibitor (112). Moreover, genistein suppressed 11β-HSD1 in rodent adipose tissue and blocked the glucocorticoid amplification (113). However, in male ob/ob mice a diet rich in genistein (600 mg/kg) for 4 weeks reduced hypercorticosteronism, decreasing protein expression of renal 11β-HSD2 without changes in hepatic 11β-HSD1 (114).

Teich and Pivovarov (115) assessed the inhibitory action on 11β-HSD1 activity of curcumin on preserving metabolic health and limiting adipose tissue growth following the cessation of daily exercise and caloric restriction (50–65% of ad libitum intake) in Sprague–Dawley rats. As main findings, the authors stated that curcumin (200 mg/kg) significantly reduced insulin, homeostasis model assessment-insulin-resistance (HOMA-IR), and C-reactive protein levels, and also exhibited inhibitory activity against human and rat 11β-HSD1 in intact cells (IC_50_ = 2.3 and 5.8 µM, respectively) and on 11β-HSD2 (IC_50_ = 14.56 and 11.92 µM, respectively) (116). Also, curcumin (200 mg/kg) reduced serum glucose, cholesterol, triglyceride, low density lipoprotein levels in high-fat-diet-induced obese rats (116).

Resveratrol is a plant-derived polyphenolic compound and a potent antioxidant. The effects of resveratrol on 11β-HSD1 activity in rodent adipose tissue was studied by Tagawa and Kubota (117). In this study, the 11β-HSD1 activity by resveratrol was inhibited (IC_50_ value of 35.2  μM) but resveratrol did not affect the activities of 11β-HSD2 and hexose-6-phosphate dehydrogenase. Several teas (*Camellia sinensis* (L.) Kuntze) and tea specific polyphenolic compounds were also tested in human liver microsomes and purified human 11β-HSD1 for their possible antidiabetic potential *via* cortisone reduction by 11β-HSD1 inhibition (118). The polyphenol (−)-epigallocatechin gallate (EGCG) exhibited the strongest inhibition of 11β-HSD1 (IC_50_  =  57.99 μM for reduction; IC_50_  =  131.2 μM for oxidation).

In a systematic review and meta-analysis of 10 short and small-size randomized controlled trials, ginger (*Zingiber officinale* Roscoe) has also demonstrated good ameliorative effects in fasting blood glycemia (FBG), insulin, HOMA-IR and HbA1c (1–3 g/day for 4–12 week, n <40) control and insulin sensitivity (119). In particular, in two studies, T2DM patients decreased FBG, HbA1c, insulin and HOMA-IR after receive a daily capsule with 1.6 or 1 g/day of ginger, respectively. In the first study, 33 subjects received the treatment and 30 were controls (60), meanwhile in the second one, the sample were 39 subjects and the control 31 (63). Moreover, specifically, the three gingerol derivatives, namely paradol, (E)-shogaol, and (5R)-acetoxy-gingerol, were able to inhibit human and mouse 11β-HSD1 activities (IC_50_ values ranged between 1.09 and 1.30 μM) (120). Licorice (*Glycyrrhiza glabra* L.) is a plant that has been increasingly studied for its antidiabetic potential (121) and has shown pre-translational inhibitory effects of 11β-HSD *in vitro* (rat pituitary GH3 cells) and *in vivo* (rats, 75 mg/kg/day glycyrrhizic for 5 days) (121).

Gumy and Thurnbichler (122) also investigated the ability of some medicinal plants extracts to inhibit 11β-HSD1 in transfected HEK-293 cells. As main findings, the authors found that leave extracts of loquat (*Eriobotrya japonica* (Thunb.) Lindl.) and extracts of roasted but not coffee beans were capable of inhibiting 11β-HSD1. Concomitantly, there have been several clinical studies exploring the effect of natural inhibitors of 11β-HSD1 in T2DM, principally soy isoflavones, despite some of them are still in process. In general, results are inconsistent, mainly because of the small sample size, different characteristics of enrolled population, source and doses of administration or the use of other molecules in combination.

There are several meta-analysis of clinical studies related to beneficial effect of flavonoids, lignans and isoflavones intake in decreasing T2DM risk or improving biochemicals parameters of glucose metabolism (123, 124). For example, a daily intake of soy product with 9 g of protein for 1 year in 323 overweight post-menopausal women (control 39,062 women), decreased glycosuria (67). Moreover, the intake of 10 mg of s-equol for 12 weeks in 49 women and men (control 49 subjects), decreased HbA1c (68). Similar effect showed the administration of 360 mg/day flaxseed-derived lignan supplement in 37 T2DM patients (control 36 subjects) in two periods of 12 weeks each one (70). In the case of genistein, the administration of 54 mg/day for 2 years in 198 post-menopausal women (control 191 women) reduced fasting insulin levels and also improved FBG and HOMA-IR (69). Also isoflavones intake showed beneficial effect in post-menopausal women, at doses of 40 and 80 g/day for a year, decreasing 5.2 and 3.3 mg/dl FBG, respectively (n = 68 each experimental group, control n = 67) (71).

Also, there are long long longitudinal studies that evaluated the beneficial effect of soy food, phytoestrogens, flavones, isoflavones and lignans consume. A small study performed in 468 men stratified according to phytoestrogen intake for two years, showed that lignan intake decreased C-peptide level, but isoflavones do not had effect (72). Other study performed in 299 pregnant women followed for 2 year of National Health and Nutrition Examination Survey-US, found an inverse relation between isoflavones intake and FBG, insulin and HOMA-IR (78). Goodman-Gruen and Kritz-Silverstein (79) analyzed the genistein intake in post-menopausal women for 1 year and founded an inverse relation with fasting insulin. Date from six cohort studies were also used to analyze the effect of soy foods, flavonoids, isoflavones and lignans. Zamora-Ros and Forouhi (77) found lower risk of T2DM associate to higher intake of flavonoids and lignans in 15,258 men and women of EPIC-InterAct study followed for a year. In date of the Nurses’ Health Study (NHS) I and II, lignan intake for 6 year was associated to lower risk of T2DM in 1,107 women over 1,107 control (80). Moreover, when soy food intake was analyzed in 43,176 men and women from Singapore Chinese Health Study, followed for 5.7 years, only isoflavones and unsweet soy food were associated to lower risk of T2DM, meanwhile sweated soy food was associate to higher risk (73). In date from Japan Public Health Center-Based Prospective Study, in 59,791 men and women followed for 5 years, consume of soy products and isoflavones reduced the risk of T2DM in overweight-obese women (76). Finally, in 64,191 post-menopausal women of Shangai Women’s Health Study, soy food reduced risk of T2DM when is consumed for 4.6 years (74).

In summary, case-control and prospective cohort studies suggest inverse associations between the total flavonoids, isoflavones and lignans intake with T2DM risk, and improvement of the disease development, with different effectiveness. Taken together, the findings evidence that there are other compounds in soy, such as lipids and fiber, than can have glycemic effect or can interact with flavone and that is necessary to measure specific phytoestrogen biomarkers to study its relationship with T2DM risk.

There are also some few clinical trials addressing the role of curcumin in T2DM. In a small meta-analysis, three out of five randomized clinical trials, it was stated a reduction in FBG, HOMA-IR and HbA1c, in doses between 250 and 1,000 mg and treatment between 10 days and 9 months (124). In the longest study (9 months, 120 treated and 120 controls), curcuminoids-treated prediabetic subjects (1.5 g/day) did not develop T2DM, meanwhile in placebo group, 16% developed T2DM (82). Treated group also showed lower C-peptide and higher HOMA-β, revealing improvements in β cell function. Moreover, in T2DM patients (50 treated and 50 controls), curcuminoids (500 mg/day for 3 months) decreased FBG, C-peptide and HbA1c (83). Other meta-analysis (125) showed that when curcumin was administrated in nano-micelles (300 mg/day for 3 months, n = 50 for treated and control T2DM subjects), FBG reduced 18% and HbA1c 11% (84), similar result were obtained when 30 mg/day were administrated for 3 months (n = 35 for treated and control T2DM groups) (81). In addition, one out of three studies that analyzed renal function in T2DM patients, found beneficial effect of curcumin (125). Curcumin has rapid metabolism and low intestinal absorption, for that, micelles, nanoparticles, liposomes and phospholipid complexes have been used in some studies to improve its bioavailability and biological efficacy (124). Moreover, a differential gender-bioavailability has been reported, related to a higher hepatic metabolization of curcumin in men and effect of body fat in women, which could be consider in future studies (126).

Finally, there are some preclinical and clinical studies exploring the effect of green and black tea in T2DM. Studies were performed between 12 weeks and 18 months with doses/day between 200 mg/day and 2.5 g/3 times for day (127). For example, in a clinical trial for 12 weeks performed in 32 T2DM patients and 28 controls, 1 g/day of green tea infusion reduced HbA1c (86, 88). But, in other four studies, no changes were observed after green tea administration when the intervention was shorter, as in Toolsee, Aruoma (85) study (4 weeks, 3 cups/day, n = 65 and control n = 58) (127). However, when three (n = 24) or 4 (n = 25) cups of green tea were administrated for 4 weeks in T2DM subject (control n = 14), systolic and diastolic blood pressure were decreased, with other four similar studies also evidencing significant improvements in both blood pressure and anthropometric data in T2DM patients (128, 129). Noteworthy, despite studies reveal controversial results related to effect of tea in T2DM, with an evident importance of the duration of the intervention, should be underlined that green tea also has flavan-3-ols, that by itself have shown beneficial effects in clinical trials with hypertense, diabetic and overweight subjects (130). Finally, few human studies relating EGCG intake with T2DM have been done, with only a clinical trial finished (25 treated and 25 control T2DM subjects) found that 300 mg of EGCG/day for 8 weeks significantly reduced FBG (90).

### Enhance Insulin Activity *via* Protein Tyrosine Phosphatase 1B Inhibition

The improvement in insulin sensitivity is a key therapeutic strategy in T2DM. Protein tyrosine phosphatase 1B (PTP1B) inhibitors improve the insulin receptor sensitivity and, in the latter years, targeting PTP1B inhibitors is being considered an attractive target to treat T2DM (131). Binding of type 1 insulin-like growth factor (IGF-1) to its tetrameric receptor induces the auto-phosphorylation of the receptor, the downstream activation of protein kinase B (PKB) and mitogen-activated protein kinases (MAPK) pathways triggering the GLUT4 transporter translocation to the plasma membrane (132).

Natural products possessing inhibitory activities against PTP1B were reviewed by Jiang and Liang (133). In their work, the authors provide an overview of approximately 300 secondary metabolites that were isolated from various natural sources or derived from synthetic process. Among them, the phytochemicals targeting PTP1B include phenolics, terpenes, steroids, N- or S-containing compounds, and miscellaneous phytochemicals.

For example, resveratrol (dose equivalent at 2.5 mg/kg orally administered *via* drinking water) improved peripheral insulin signaling independently of Sirt1 in diabetic mice in association with PTP1B inhibition (134). Sirt1 is one of seven mammalian orthologs of the yeast protein, and has been suggested to be involved in the processes of glucose homeostasis and insulin secretion (135). Indeed, several studies have shown association of the effect of resveratrol in both proteins together (PTP1B and Sirt1) (136, 137).

Chuang and Martinez (138) showed that quercetin is more effective than resveratrol in inhibiting PTP1B in primary cultures of human adipocytes treated with TNF-α. Indeed, the treatment with quercetin decreased the mRNA levels of PTP1B, while resveratrol treatment had no effect on PTP1B expression (138).

Curcumin and cinnamaldehyde decreased PTP1B enzymatic activity in breast cancer MCF-7 cell line (139), despite curcumin was more effective than cinnamaldehyde. Briefly, curcumin inhibited PTP1B at concentrations starting from 1 μM (IC_50_ ≈ 100 μM) (139). In fructose-fed rats, curcumin ingestion inhibited PTP1B and improved insulin and leptin sensitivity in the liver of rats, also protecting hypertriglyceridemia and hepatic steatosis induced by fructose diet (140). Also, it has been suggested that curcumin activate miRNA206 and then improve insulin sensibility (141).

Papaverine from *Papaver somniferum* L. is an isoquinoline alkaloid with inhibitory effects against PTP1B in humans (142). *In vitro* papaverine illustrated potent *in vitro* inhibitory effects against recombinant h-PTP1B (IC_50_ = 1.20 µM), while *in vivo* it led to a marked decreased in fasting blood glucose level in Balb/c mice (142).

Xu and Wang (143) studied anti-diabetic effect of bis(2,3-dibromo-4,5-dihydroxybenzyl) ether (BDDE), a bromophenol isolated from the red alga (*Odonthalia corymbifera*). The *in vitro* treatment with 2.5, 5, or 10 μM of BDDE for 16 h dose-dependently inhibited the over-expression of PTP1B in insulin-resistant HepG2 cells. *In vivo* in db/db mice model, the BDDE activity was compared with that of metformin, where a significant decrease in blood glucose levels and HbA1c were stated following BDDE treatment, without weight gain (143).

Inhibitory activity of lipophilic compounds from *Salvia miltiorrhiza* Bunge roots against PTP1B, was also investigated (144), being shown PTP1B inhibitory effects using cryptotanshinone (IC_50_ = 5.5 ± 0.9 µM), tanshinol B (IC_50_ = 4.7 ± 0.4 µM) and dehydrodanshenol (IC_50_ = 8.5 ± 0.5 µM) (144). *In vivo* studies in diabetic rats treated with a polyphenolic fraction of *S. miltiorrhiza*, also evidenced a lower fasting glucose (145). In this way, the active ingredient tanshinone IIA reduced glycemia in fasted mice after an acute injection of a glucose bolus (146).

There is limited data from human assays exploring specific targeting PTP1B inhibitors, and that available investigated the effect of resveratrol. The first clinical trial, composed of a very small sample (total 19, 10 intervened and nine controls), found that 10 mg/day of resveratrol capsule administered for a month improved HOMA-IR (91) meanwhile in other, performed in 28 medicated T2DM subjects treated with 250 mg/day of resveratrol for 3 months improved HbA1c level (control n = 29) (92). However, no effect of resveratrol was observed in three studies, in 15 post-menopausal women (control n = 14), 10 overweight men with no-alcoholic liver disease (control n = 14) and 12 obese men (control n = 12) when was administrated in doses of 75 g/day for 12 weeks (93), 3,000 mg/day for 8 weeks (94) and 300 mg/day for 4 weeks (95), respectively. In preliminary date from PREDIMED study (Prevention with Mediterranean Diet) 1,000 obese T2DM patients followed for two years improved FBG (96). Data of resveratrol clinical trials is resumed in **Table 1**. In general, the beneficial preventive or curative effect of resveratrol in T2DM depend on doses, time of intervention and characteristic of population, but as other phytochemicals, should be consumed as supplement, because the doses ingested through diet are very low. Moreover, urinary metabolites should be controlled (130, 147, 148).

### Estradiol Regulation Via 17β-Hydroxysteroid Dehydrogenase

Estradiol induces metabolic homeostasis and its accumulation in serum may be indicative of estrogen resistance, metabolic deficiency and T2DM (149). In postmenopausal women, elevated levels of circulating estradiol were associated with T2DM (150) but did not reflected causality. 17β-HSDs play a key role in both degradation and activation of androgens and estrogens. Specifically, 17β-HSD1 facilitates the reduction of estrone to estradiol, while 17β-HSD2 mediates the oxidation of estradiol to estrone (151). In humans, 17β-HSD1 is expressed in placenta, ovary and breast epithelial cells, and 17β-HSD2 in placenta, lung, liver, pancreas, kidney, prostate, colon, small intestine, endometrium and breast epithelial cells (151).

Stupans and Stretch (152) examined the ability of complex phenols from olive oil to inhibit human hepatic microsomal both reductive and oxidative 17β-HSD activity. Specifically, dihydroxybenzoic acid, gallic acid, hydroxytyrosol, and oleuropein glycoside inhibited the reductive 17β-HSD activity but not the oxidative one. Rather, gallic acid stimulated the activity by approximately 30% (152).

Some molecules, like epicatechin may also activate 17β-HSDs in rat testicular Leydig cells (153). In the same way, resveratrol also regulates protein and mRNA expression of 17β-HSD in rats (154, 155). Curcumin and quercetin increased the activity of 17β-HSD, but curcumin evidenced a slightly better activity as compared to quercetin (156). On the contrary, soy isoflavones treatment in female rats decreases 17β-HSD levels (157). In this way, a previous report indicates that genistein can inhibit 17β-HSD enzyme in both human and rat testis microsomes (158).

There are few studies in human related to extra-virgin olive oil consume and T2DM (130). In two intervention studies, 500 mg of leaf extract improved insulin resistance parameters. The first study, performed in 21 overweight men treated for 12 weeks compared with 22 control men, improved β-cell function and insulin levels. Those parameters showed a higher improve when 36 subjects treated with hypolipemiant or anti-hypertensive medication were excluded (97). In another study, the intake of one capsule a day (51.1 mg oleuropein and 9.7 mg hydroxytyrosol for 12-week) improved pancreatic β-cell function and insulin sensitivity in T2DM patients. The other study was performed in 41 T2DM men for 14 week (control n = 38) and showed an improvement of HbA1c and fasting insulin levels (98). The doses of olive leaf extract administrated had 51.1 mg oleuropein and 9.7 mg hydroxytyrosol. In contrast, in other study, no significant effect was stated following administration of two polyphenol-rich olive oil (20 ml/day, 6 mg hydroxityrosol) for 6 weeks in 63 healthy participants (group 1 n = 39, group 2 n = 29, control n = 9) (99). In yet another study performed in 11 overweight T2DM adults, found that the administration of 25 ml of high phenol olive oil for 4 weeks reduced HbA1c and FBG (159). Finally, the PREDIMED trial revealed that Mediterranean diet supplemented with extra-virgin olive oil reduced in 40% the T2DM incidence while improved glucose metabolism in participants with high cardiovascular risk (100, 160). Also 50 ml of virgin olive oil a day increased expression of insulin-related candidate genes in peripheral mononuclear cells (161). Indeed, extra-virgin olive oil is not only rich in polyphenols, but also has flavonoids, flavones and lignans, that present action over glucose metabolism, that can be considered when data from intervention studies is analyzed.

### Incoming Glucose Into Hexosamine Biosynthesis Regulation *via* Glutamine-Fructose-6-Phosphate Aminotransferase

Glutamine-fructose-6-phosphate aminotransferase (GFAT) is a rate-limiting enzyme in the hexosamine biosynthetic pathway that plays an important role in T2DM (162). GFAT enzyme converts fructose-6-phosphate to glucosamine-6-phosphate. In mammals, the glucose integration through the hexosamine biosynthetic pathway is regarded as a cell nutrient sensor and this pathway is one of the mechanisms through which hyperglycemia mediates peripheral insulin resistance (163) and diabetic complications (164). In this sense, an enhanced activity of human GFAT has been implicated in insulin resistance in cellular and animal models (165, 166).

In Wistar rats, fenugreek (*Trigonella foenum-graecum* L.) was revealed to be able to control the increase in GFAT activity provoked by corn starch diet and reduced kidney damage (167). Similarly, *Coriandrum sativum* L. fruit and its phytoconstituents evaluated in an evaluated *in silico* model (168), evidenced, specifically the compound limonene, to be a good inhibitor of GFAT. Indeed, a meta-analysis including 10 clinical trials revealed that fenugreek ingest between 1 and 100 g/day for 10 to 84 days reduced FBG by −17.93 mg/dl, 2 h post-load glucose by −39.46 mg/dl and HbA1c by −0.85%. Fenugreek was administered in powder, alcoholic extract or capsules (169). More prominent changes were found in T2DM and overweight patients (20 case and 20 control subjects, 1 g/day for 2 weeks) (102, 169), and T2DM with coronary arterial disease (30 case and 30 control subjects, 25 g/twice daily for 1 week) (101, 103). It has been suggested that larger double blind randomized trials should be performed according to rigorous standards for herbal intervention, because the trails analyzed and available so far have low sample sizes (≤25 subjects) (169). Another meta-analysis analyzing the same articles was performed later, with similar conclusions, highlighting therefore the low quality of studies and underlining the promising beneficial effects of fenugreek in pre-diabetes subjects (170). Another later simple study in 60 metformin-treated T2DM patients found that thrice daily 1 g fenugreek for 12-week improved FBG, post-prandial glucose and HbA1c in higher proportion when compared to the group receiving only metformin (n = 30 each group), suggesting that fenugreek can be used as complementary therapy in T2DM control (104). Meanwhile another longitudinal study found that in T2DM patient with hyperglycemia and under different treatments, 25 g/day of fenugreek for 24 week decreased glucosuria and HbA1c (n = 60, 10 control) (105).

### Insulin Secretion, Glucose Uptake and Gluconeogenesis Regulation *via* Mono-ADP-Ribosyltransferase-Sirtuin-6

Sirtuin 6 (SIRT6) has both NAD^+^-dependent deacetylase (171) and mono-ADP-ribosyltransfease activity (172), and is targeted by antidiabetic epidrugs exhibiting inhibitory or activating mechanisms. Briefly, the absence of SIRT6 has been linked to an increased tissue glucose uptake and decreased serum glucose levels (173) through inhibiting the expression of the transcription factor hypoxia inducible factor (HIF)-1α, which is involved in the transcription of glucose transporters (174). Also, SIRT6 boosts the deacetylation of peroxisome proliferator-activated receptor-γ coactivator (PGC)-1α, which robustly stimulates hepatic gluconeogenesis (175).

The interactions between the main bioactive compounds of ginger (i.e., 4-gingerol, 6-gingerol, 8-gingerol, 10-gingerol, 6-shogaol, and β-bisabolol) and protein targets (GFAT, SIRT6, GLUT4, 11β-HSD1 and glycogen phosphorylase) were studied using computational interaction methods, molecular docking and pharmacophore (176). SIRT6 and GFAT showed binding affinity ranges lower than 11β-HSD1 and glycogen phosphorylase but more stables, and strong interaction with GLUT4 was observed. The study concluded that the synergistic mixture of ginger phytochemicals may have functional effects for T2DM treating therapy.

*Euphorbia thymifolia* L. is a medicinal plant with reported anti-hyperglycemic activity (177). In a study aiming to investigate the antidiabetic mechanism involved, molecular interactions between phytochemicals in *E. thymifolia* and protein targets (11β-HSD1, GFAT, PTP1B, and SIRT6) were analyzed (178). As main findings, seven bioactive compounds with high binding affinity (<−8.0 kcal/mol) to all four targeted proteins were found, namely β-amyrine, taraxerol, 1-*O*-galloyl-β-D-glucose, corilagin, cosmosiin, quercetin-3-galactoside and quercitrin. To date, there is no data available on clinical trials exploring specific targeting SIRT6 inhibitors, so that this should be an area of high search in the near future.

## Conclusion

Epidrugs are another strategy to prevent or delay the T2DM onset through epigenetic mechanisms. The use of these new drugs has shown to have a high potential since they can genetically modulate diseases while other treatments act through other biochemical mechanisms. Many known plants (i.e. ginger, tea and fenugreek) and phytochemicals (i.e. curcumin and resveratrol) with an impact on diabetes have been shown to interact with different protein targets of T2DM. However, in some cases, there are controversial results. Data currently available underline that some bioactive compounds have epigenetic regulatory effects and appear to be useful for therapeutic and/or complementary purposes along with pharmacological hypoglycemic drugs, featured by numerous side effects. The mechanisms involved in the therapeutic effects of the potential epidrugs discussed in this review are the decrease of insulin resistance *via* 11β-HSDs inhibition, the enhance of insulin activity *via* PTP1B inhibition, estradiol regulation *via* 17β-HSD inhibition, incoming glucose into hexosamine biosynthesis regulation *via* GFAT inhibition, and the role of SIRT6 in insulin secretion, glucose uptake and gluconeogenesis regulation. Further, increasingly deeper studies on natural and synthetic compounds and new protein targets must be performed. In addition, new preclinical studies must also be done to better elucidate the epigenetic mechanisms, along with new clinical studies to determine the effectiveness of these epidrugs.

## Author Contributions

AA-H, NC-M, JS-R, and MM, critically reviewed the manuscript. All authors contributed to the article and approved the submitted version.

## Funding

This work was supported by CONICYT PIA/APOYO CCTE AFB170007.

## Conflict of Interest

The authors declare that the research was conducted in the absence of any commercial or financial relationships that could be construed as a potential conflict of interest.
